# Long Non-Coding RNA MALAT1 Regulates HMOX1 in Sickle Cell Disease-Associated Pulmonary Hypertension

**DOI:** 10.3390/cells15020154

**Published:** 2026-01-15

**Authors:** Viranuj Sueblinvong, Sarah S. Chang, Jing Ma, David R. Archer, Solomon Ofori-Acquah, Roy L. Sutliff, Changwon Park, C. Michael Hart, Benjamin T. Kopp, Bum-Yong Kang

**Affiliations:** 1 Department of Medicine, Division of Pulmonary, Allergy, Critical Care, and Sleep Medicine, Emory University School of Medicine, Atlanta, GA 30322, USA; vsuebli@emory.edu (V.S.); sarah.s.chang@emory.edu (S.S.C.); jma22@emory.edu (J.M.); charles.hart3@va.gov (C.M.H.); 2 Atlanta Veterans Affairs Healthcare System, Decatur, GA 30033, USA; 3 Department of Pediatrics, Division of Hematology, Oncology, and BMT, Emory University School of Medicine, 2015 Uppergate Drive, Atlanta, GA 30322, USA; darcher@emory.edu (D.R.A.); solomon.fiifi.ofori-acquah@emory.edu (S.O.-A.); 4 National Heart, Lung and Blood Institute, National Institutes of Health, Bethesda, MD 20892, USA; roy.sutliff@nih.gov; 5 Department of Molecular and Cellular Physiology, Louisiana State University Health Science Center, Shreveport, LA 71103, USA; changwon.park@lsuhs.edu; 6 Department of Pediatrics, Division of Pulmonology, Asthma, Cystic Fibrosis, and Sleep Medicine, Emory University School of Medicine, 2015 Uppergate Drive, Atlanta, GA 30322, USA; benjamin.t.kopp@emory.edu

**Keywords:** lncRNA, MALAT1, sickle cell disease, pulmonary hypertension, endothelial dysfunction

## Abstract

Pulmonary hypertension (PH) causes morbidity and mortality in sickle cell disease (SCD). The release of heme during hemolysis triggers endothelial dysfunction and contributes to PH. Long non-coding RNAs (lncRNAs) may play a pivotal role in endothelial dysfunction and PH pathogenesis. This study assessed the regulatory role of the lncRNA–heme oxygenase-1 (HMOX1) axis in SCD-associated PH pathogenesis. Total RNAs were isolated from the lungs of 15–17-week-old sickle cell (*SS*) mice and littermate controls (*AA*) mice and subjected to lncRNA expression profiling using the Arrystar™ lncRNA array. Volcano plot filtering was used to screen for differentially expressed lncRNAs and mRNAs with statistical significance (fold change > 1.8, *p* < 0.05). A total of 3915 lncRNAs were upregulated and a total of 3545 lncRNAs were downregulated in the lungs of *SS* mice compared to AA mice. To validate differentially expressed lncRNAs, six upregulated lncRNAs and six downregulated lncRNAs were selected for quantitative PCR. MALAT1 expression was significantly upregulated in the lungs of *SS* mice and in hemin-treated human pulmonary artery endothelial cells (HPAECs), suggesting that hemolysis induces MALAT1. Functional studies revealed that MALAT1 depletion increased, while MALAT1 overexpression decreased, the endothelial dysfunction markers endothelin-1 (ET-1) and vascular cell adhesion molecule-1 (VCAM1), indicating a protective role of MALAT1 in maintaining endothelial homeostasis. In vivo, adenoviral MALAT1 overexpression attenuated PH, right ventricular hypertrophy (RVH), vascular remodeling, and reduced ET-1 and VCAM1 expression in *SS* mice. Given that HMOX1 protects endothelial cells during hemolysis, we observed that HMOX1 expression and activity were elevated in *SS* mouse lungs and hemin-treated HPAECs. HMOX1 knockdown enhanced ET-1 and VCAM1 expression, confirming its endothelial-protective function. Importantly, MALAT1 overexpression increased HMOX1 expression and activity, whereas MALAT1 knockdown reduced HMOX1 levels and mRNA stability. Collectively, these findings identify MALAT1 as a protective regulator that mitigates endothelial dysfunction, vascular remodeling, and PH in SCD, at least in part through the induction of HMOX1. These results suggest that SCD modulates the MALAT1–HMOX1 axis, and further characterization of MALAT1 function may provide new insights into SCD-associated endothelial dysfunction and PH pathogenesis, as well as identify novel therapeutic targets.

## 1. Introduction

Pulmonary hypertension (PH) is a recognized complication of sickle cell disease (SCD) associated with high morbidity and mortality, regardless of PH severity [1,2,3]. SCD is characterized by chronic intravascular hemolysis that generates excess heme in the blood and recurrent vaso-occlusive insults associated with painful crisis, acute chest syndrome, and chronic organ damage and failure [4]. Intravascular hemolysis activates the pulmonary endothelium, which in turn upregulates hemin activating adhesion molecule expression, such as vascular endothelial cell adhesion molecule 1 (VCAM1), leading to endothelial dysfunction [5]. Endothelial dysfunction caused by hemolysis represents a fundamental derangement that is central to end-organ dysfunction in SCD-associated PH (SCD-PH) [6]. Current therapies for SCD-PH that target vascular dilation do not reverse the abnormal, proliferative, vascular cell phenotypes that underlie the pulmonary vasculopathy in this disorder. These considerations indicate that new therapeutic approaches are needed. For example, endothelin-1 (ET-1) is elevated in both SCD and PH patients [7,8,9,10,11,12,13]. ET-1 causes endothelial dysfunction, vasoconstriction, vascular cell migration, and proliferation in PH [8,14]. However, clinical trials in SCD-PH examining the ET-1 receptor antagonist, bosentan, produced insignificant decreases in pulmonary vascular resistance and conflicting results [15]. Therefore, the current study seeks to address the complex pathobiology of SCD-PH by examining novel targetable molecular mechanisms of endothelial dysfunction in SCD-PH.

Emerging evidence suggests that long non-coding RNAs (lncRNAs) play important roles in regulating gene expression in multiple critical cellular pathways including regulation of uncontrolled cell proliferation, cell growth, and apoptosis [16,17,18,19]. LncRNA can promote or inhibit gene expression at the level of mRNA transcription, translation, RNA stabilization, and through their ability to interact with microRNAs (miRNAs) [20]. Moreover, mounting evidence demonstrates that lncRNAs play a significant role in the pathobiology of PH and may serve as viable molecular targets for therapy [21,22]. For example, several lncRNAs have been implicated in PH pathogenesis. Specifically, lncRNA metastasis-associated lung adenocarcinoma transcript 1 (MALAT1) is highly conserved in humans and mice and abundantly expression in various cells and tissues [23,24]. MALAT1 has many reported roles in vascular pathology, including its involvement in endothelial cell activation, inflammatory signaling, and vascular remodeling in other disease contexts. Interestingly, we found MALAT1 expression was upregulated in *SS* mouse lungs. However, the specific contribution of MALAT1 to endothelial dysfunction in SCD-PH remains poorly understood and the role of MALAT1 in endothelial cell function has not been elucidated in preclinical SCD-PH rodent models.

Heme oxygenase 1 (HMOX1) is an essential enzyme that degrades heme released during intravascular hemolysis and promotes endothelial health through antioxidant, cytoprotective, and heme catabolic pathways. HMOX1 also protects endothelial function in SCD [25]. In relation to PH, HMOX-1 expression is reduced in the lungs of patients with PH, while knockout of HMOX1 exacerbates hypoxia-induced PH and right ventricular hypertrophy (RVH) in mice [26]. Furthermore, HMOX1 overexpression in alveolar epithelial cells decreased hypoxia-induced PH [27]. Intriguingly, increased HMOX1 levels have been observed in neutrophils, mononuclear cells [28], and tissues of patients and transgenic mice with SCD [29]. However, the mechanisms and functional significance underlying these HMOX1 increases via lncRNAs in SCD have not been defined. Based on our observation that MALAT1 is upregulated in SCD models and its known roles in endothelial activation and stress responses, we hypothesized that MALAT1 contributes to endothelial dysfunction in SCD-PH by regulating HMOX1 expression, representing a novel MALAT1–HMOX1 regulatory axis in vascular pathology.

In this study, we report a novel link between lncRNA MALAT1 and HMOX1 in SCD-PH and that MALAT1 could serve as an effective therapeutic target in SCD-PH.

## 2. Materials and Methods

### 2.1. Sickle Mouse Lung Tissues

Lung tissues were isolated from littermate controls (*AA*) mice and from the Townes humanized mouse model of SCD (*SS*) (JAX:013071) [30].

### 2.2. Human Pulmonary Artery Endothelial Cells (HPAECs)

HPAECs (passages 2–6, ScienCell, Carlsbad, CA, USA) were incubated with 5 µM hemin (HEM) or dimethyl sulfoxide (0.1% DMSO), used as control [CON] for up to 72 h as previously reported [31].

### 2.3. Assessment of PH in MALAT1-Overpressing Sickle Cell Mice

Selected *AA* or *SS* mice (15–17 weeks old) were treated with control green fluorescent protein (GFP) or adenoviral MALAT1 (AdMALAT1, 25 multiplicity of infection (MOI), Supplementary Figure S1A) particles using intranasal injection as previously reported [31]. On day 14 following transfection, mice were subjected to measurements of right ventricular systolic pressure (RVSP) and right ventricular hypertrophy (RVH) as reported [32]. All experiments using mice were approved by the Institutional Animal Care and Use Committee of the Atlanta Veterans Affairs Medical Center and were conducted in accordance with institutional standards for the humane treatment of laboratory animals.

### 2.4. Reagents

HPAECs were obtained from ScienCell Research Laboratories (Carlsbad, CA, USA). HMOX1 antibody was purchased from Santa Cruz Biotechnology (Santa Cruz, CA, USA). The following reagents were obtained from Sigma-Aldrich (St. Louis, MO, USA): GAPDH antibody, hemin, fetal bovine serum (FBS), dimethyl sulfoxide (DMSO), and actinomycin D.

### 2.5. Cell-Based Enzyme-Linked Immunosorbent Assay (ELISA)

To measure HMOX1 activity in HPAECs, an In-Cell ELISA was performed and normalized to GAPDH levels within the same well using the Odyssey Imaging System (LI-COR Biosciences, Lincoln, NE, USA), according to the manufacturer’s instructions.

### 2.6. MALAT1 Loss or Gain of Function

For MALAT1 loss-of-function experiments, HPAECs were transfected with MALAT1 antisense oligonucleotide (ASO) or appropriate control (1 nM, Qiagen, Germantown, MD, USA) using Lipofectamine 3000 transfection reagent (Thermo Fisher Scientific, Waltham, MA, USA), according to the manufacturer’s instructions. At 6 h following transfection, the transfection medium was replaced with endothelial cells growth medium (EGM) containing 5% FBS, and cells were incubated at room temperature for 72 h. Selected HPAECs were treated ± HEM (5 μM) or DMSO directly to the culture medium for up to 72 h. HPAEC lysates were harvested and analyzed for MALAT1, ET-1, VCAM1, and HMOX1 levels by qRT-PCR.

To overexpress MALAT1, HPAECs were transfected with adenovirus-mediated MALAT1 (AdMALAT1, 5 MOI, Supplementary Figure S1B) or green fluorescent protein (GFP)-tagged control adenovirus particles, as reported [33]. At 6 h following transfection, media were replaced with fresh 5% FBS EGM, and HPAECs were treated with DMSO or HEM (5 µM) for 72 h. HPAEC lysates were then harvested and examined for MALAT1, ET-1, VCAM1, and HMOX1 levels using qRT-PCR analysis.

### 2.7. HMOX1 siRNA

For HMOX1 loss-of-function, HPAECs were transfected with scrambled or HMOX1 RNAi duplexes (10 nM, Integrated DNA Technologies, Coralville, IA, USA) using Lipofectamine 3000 transfection reagent (Invitrogen, Carlsbad, CA, USA), according to the manufacturer’s instructions. After 6 h of transfection, the transfection medium was replaced with EGM containing 5% FBS, and cells were incubated at room temperature for 72 h. To determine whether depletion of HMOX1 affects its activity, the In-Cell ELISA was performed. Furthermore, HPAEC lysates were harvested and analyzed for ET-1 and VCAM1 levels using qRT-PCR.

### 2.8. Hematoxylin and Eosin (H&E) Staining

To examine pulmonary vascular remodeling in mouse small arterioles, lung sections were stained with hematoxylin and eosin (H&E) according to the manufacturer’s instructions (Abcam, Cambridge, MA, USA). For each lung, sections from 10 distinct tissue blocks were analyzed. Images were captured using Keyence imaging system (Itasca, IL, USA).

### 2.9. Messenger RNA Stability Assay

To inhibit de novo HMOX1 mRNA synthesis, HPAECs were transfected with ASO control or ASO MALAT1 and incubated for 72 h, and then 5 μg/mL actinomycin D (Apexbio, Houston, TX, USA) was treated time-dependently. Total RNAs were isolated using mirVana kit (Thermo Fisher Scientific), and HMOX1 mRNA levels were measured by qRT-PCR analysis. HMOX1 mRNA half-life was determined by comparing its level at each time point to the baseline level before actinomycin D treatment.

### 2.10. Messenger RNA Quantitative Real-Time Polymerase Chain Reaction (qRT-PCR) Analysis

To measure MALAT1, ET-1, VCAM1, and HMOX1 levels, total RNAs were isolated from mouse lungs and HPAECs using the mirVana kit (Thermo Fisher Scientific). MALAT1, ET-1, VCAM1, and HMOX1 mRNA levels were quantified by qRT-PCR using human and mouse primers (Table 1), as previously described [34,35]. GAPDH mRNA levels were used as a control.

### 2.11. Western Blot Analysis

Homogenates from mouse lungs or from hemin-treated HPAECs were subjected to Western blot analysis, as previously reported [34]. The primary HMOX1 mouse monoclonal antibody (1:500 dilution, Cat # SC-136960, 32 kDa) was purchased from Santa Cruz Biotechnology (Santa Cruz, CA, USA). GAPDH rabbit polyclonal antibody (1:10,000 dilution, Cat # G9545, 37 kDa) was purchased from Sigma-Aldrich (St. Louis, MO, USA). Relative protein levels were visualized using Li-Cor Odyssey DLx Image Studio^TM^ 6.1 software, quantified Image J software 2.2.0-beta6, and normalized to GAPDH levels within the same lane.

### 2.12. Statistical Analysis

All data are presented as mean ± standard error of the mean (SE). Shapiro–Wilk test was used to assess normality. For data that were not normally distributed, the Mann–Whitney U test was used to compare two independent groups, and the Kruskal–Wallis analysis of variance (ANOVA) was used to compare three or more independent groups. *Post hoc* analyses were performed using the Student Neuman Keuls test to detect differences between specific groups. In studies comparing only two experimental groups, data were analyzed with Student’s *t*-test to determine the significance of treatment effects. The level of statistical significance was taken as *p* < 0.05. Statistical analyses were carried out using GraphPad Prism, Version 8.0 software (LaJolla, CA, USA).

## 3. Results

### 3.1. MALAT1 Expression Is Increased in SS Mouse Lungs and in Hemin-Treated HPAECs

**Figure 1:**
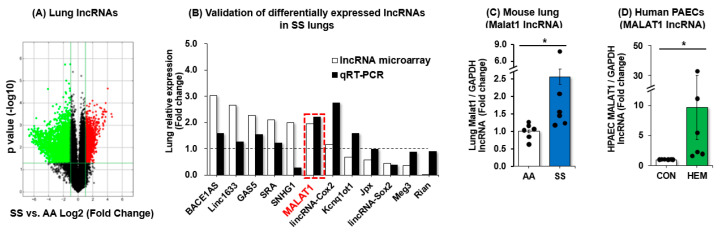
Hemolysis induces MALAT1 levels in vivo and in vitro. Whole lung homogenates were collected from littermate control (*AA*) and sickle cell (*SS*) mice at age 15–17 weeks. (**A**) Total RNAs were subjected to lncRNA expression profiling using the Arrystar™ lncRNA array. Raw signal intensities were normalized in quantile method by GeneSpring GX software 14.9. Volcano plot filtering was used to screen for differentially expressed lncRNAs and mRNAs with statistical significance (fold change > 1.8, *p* < 0.05). Red dots indicate significantly upregulated lncRNAs, and green dots indicate downregulated lncRNAs. (**B**) Selected lncRNAs from *SS* lung were subjected to qRT-PCR for validation of microarray data. MALAT1 is highlighted in red to indicate concordance of microarray and qRT-PCR analyses. (**C**) *AA* and *SS* mouse lung MALAT1 levels were measured with qRT-PCR and expressed relative to lung GAPDH. * *p* < 0.05 vs. *AA*, *n* = 6. (**D**) HPAECs were treated with dimethyl sulfoxide vehicle (CON) or hemin (HEM, 5 µM) for 72 h. Mean HPAEC MALAT1 levels were measured with qRT-PCR. All bars represent the mean MALAT1 levels relative to GAPDH ± SE as fold-change vs. CON. * *p* < 0.05 vs. CON, *n* = 6.

To define differentially expressed lncRNAs and molecular mechanisms of lncRNAs, we examined an unbiased lncRNA microarray in *SS* mouse lungs (Arraystar^TM^). The microarray study revealed that 3915 lncRNAs were upregulated (red) and 3545 lncRNAs were downregulated (green) in lungs of *SS* mice compared to *AA* mice (Figure 1A). A full listing of all up- and down-regulated lncRNAs identified in our microarray analysis is shown in Supplementary Table S1. To validate the differentially expressed lncRNAs, we selected six biologically relevant and highly upregulated or downregulated conserved lncRNAs for quantitative analyses. Among 12 lncRNAs which were assessed in total, MALAT1 was consistently upregulated in both lncRNA microarray and qRT-PCR analysis in *SS* lungs (Figure 1B). In parallel, we found that MALAT1 is induced in *SS* mouse lungs (Figure 1C) and in hemin-treated HPAECs (Figure 1D) suggesting that hemolysis might play a role in inducing MALAT1 expression. Therefore, we further explored whether intravascular hemolysis in SCD stimulates MALAT1 expression to protect against PH and endothelial dysfunction.

### 3.2. MALAT1 Regulates Expression of Endothelial Dysfunction Markers ET-1 and VCAM1 In Vitro

**Figure 2:**
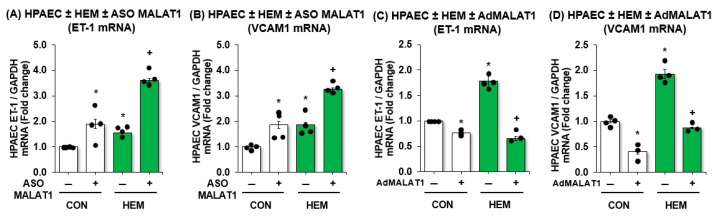
MALAT1 inversely mediates ET-1 and VCAM1 expression. HPAECs were treated with scrambled ASO or ASO MALAT1 (25 nM) for 6 h then treated with dimethyl sulfoxide vehicle (CON) or hemin (HEM, 5 µM) for an additional 72 h. Mean HPAEC ET-1 (**A**) or VCAM1 (**B**) levels were measured with qRT-PCR. All bars represent the mean mRNA levels relative to GAPDH ± SE expressed as fold-change vs. CON/ASO MALAT1(-). * *p* < 0.05 vs. CON/ASO MALAT1(-), + *p* < 0.05 vs. HEM/ASO MALAT1(-), *n* = 4. In parallel, HPAECs were treated with scrambled AdGFP or AdMALAT1 particles (5 MOI) for 6 h then treated with dimethyl sulfoxide vehicle (CON) or hemin (HEM, 5 µM) for an additional 72 h. Mean HPAEC ET-1 (**C**) or VCAM1 (**D**) levels were measured with qRT-PCR. All bars represent the mean mRNA levels relative to GAPDH ± SE expressed as fold-change vs. CON/AdMALAT1(-). * *p* < 0.05 vs. CON/AdMALAT1(-), + *p* < 0.05 vs. HEM/AdMALAT1(-), *n* = 3–4.

To investigate the role of MALAT1 in the pathogenesis of SCD-PH, we examined its functional impact on endothelial behavior in HPAECs. We first examined whether loss of MALAT1 can lead to endothelial dysfunction in hemin-treated HPAECs, simulating a chronic hemolytic state. We showed that ASO-mediated depletion of MALAT1 in HPAECs increased the expression of markers of endothelial cell (EC) dysfunction, including ET-1 (Figure 2A) and VCAM1 (Figure 2B). Furthermore, depletion of MALAT1 augmented ET-1 and VCAM1 expression in hemin-treated HPAECs (Figure 2A,B). In parallel, we examined whether gain of MALAT1 could protect HPAECs against hemin-induced endothelial dysfunction using adenovirus-mediated MALAT1 (AdMALAT1) particles. We found that MALAT1 overexpression attenuated ET-1 (Figure 2C) and VCAM1 expression (Figure 2D). MALAT1 overexpression effectively protected HPAECs from hemin-induced ET-1 and VCAM1 expression (Figure 2C,D). Collectively, these results support the notion that MALAT1 plays a protective role in endothelial function, and MALAT1 treatment may attenuate EC dysfunction in hemin-treated HPAECs.

### 3.3. MALAT1 Overexpression Reduces PH, RVH, and Vascular Remodeling with Downregulation of Endothelial Cells Dysfunction Markers, ET-1 and VCAM1

**Figure 3:**
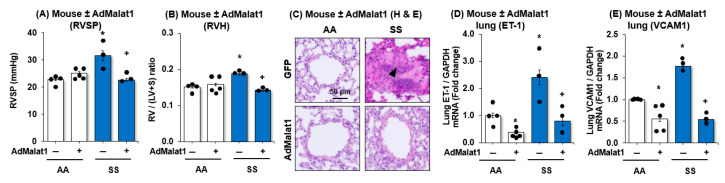
MALAT1 overexpression attenuates RV systolic pressure, RVH, vascular remodeling, and endothelial dysfunction markers ET-1 and VCAM1 gene expressions. *AA* and *SS* mice were infected with scrambled AdGFP or AdMALAT1 particles. At day 14 following transfection, mice were subjected to measurements of (**A**) right ventricular systolic pressure (RVSP) and (**B**) right ventricular hypertrophy (RVH). RVSP was measured using a 0.8 F micro-tip pressure transducer. Mice were anesthetized with isoflurane, and the transducer was inserted into the right jugular vein and advanced into the right ventricle. Right ventricular pressure was continuously recorded for 10 min and analyzed using the PowerLab system. RVH was evaluated by calculating the ratio of right ventricle weight to left ventricle plus septum weight (Fulton Index). Following euthanasia, hearts were excised, and the right ventricle was carefully separated from the left ventricle and septum to determine the weight ratio. Each bar represents mean RVSP or RVH ± SE in mmHg. *n* = 3–5. (**C**) Lung sections were stained with hematoxylin and rosin (H&E) staining according to the manufacturer’s instructions. For each lung, sections from 10 separate tissue blocks were analyzed. Images were captured using Keyence imaging system. The filled triangle indicates pulmonary vascular remodeling in the pulmonary artery wall. The resulting lungs were imaged and subjected to qRT-PCR analysis. Mean ET-1 (**D**) or VCAM1 (**E**) levels examined as fold-change vs. *AA*/AdMALAT1(-). * *p* < 0.05 vs. *AA*/AdMALAT1(-), + *p* < 0.05 vs. *SS*/AdMALAT1(-), *n* = 3–5.

We previously reported that SCD-PH is associated with vascular remodeling and endothelial dysfunction [31,35]. To determine the functional significance of MALAT1 in SCD-PH, we examined if gain of MALAT1 function regulates SCD-PH, RV hypertrophy (RVH), vascular remodeling, and markers of endothelial dysfunction. As shown in Figure 3, AdMALAT1 in *SS* mouse lungs attenuated SCD-PH as seen by a reduction in RV systolic pressure (RVSP; Figure 3A), RVH (Figure 3B), vascular remodeling (Figure 3C), ET-1 (Figure 3D), and VCAM1 levels (Figure 3E). Collectively, the data presented in Figure 3 support our hypothesis that MALAT1 plays a protective role in vascular remodeling in SCD-PH, and MALAT1 treatment may attenuate EC dysfunction, vascular remodeling, and PH.

### 3.4. HMOX1 Protects Endothelial Function in SCD-PH

**Figure 4:**
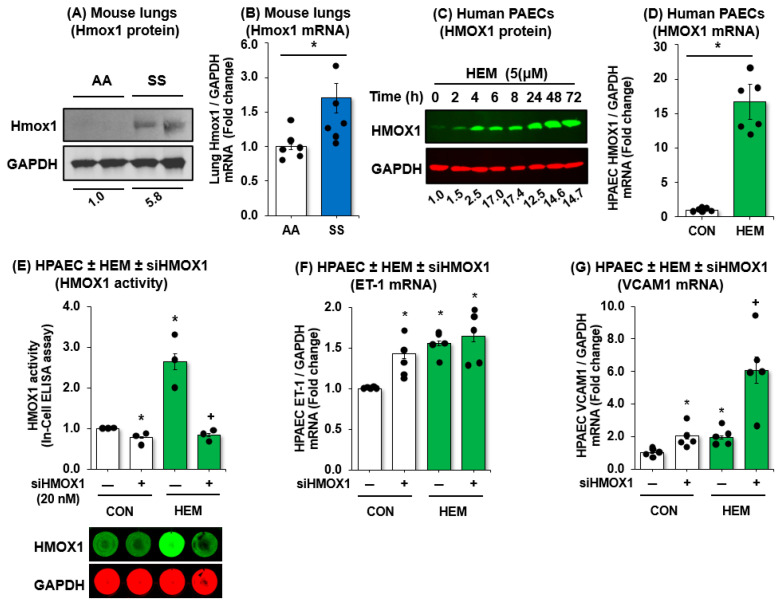
HMOX1 protects endothelial function in SCD-PH. (**A**,**B**) Whole lung homogenates were collected from littermate control (*AA*) or sickle cell (*SS*) mice at age 15–17 weeks. Mean HPAEC HMOX1 protein (**A**) or mRNA (**B**) were measured with Western blots or qRT-PCR, respectively. Each bar represents the mean ± SE expressed as fold-change vs. *AA*. * *p* < 0.05 vs. *n* = 6. (**C**,**D**) HPAECs were treated with dimethyl sulfoxide vehicle (CON) or hemin (HEM, 5 µM) for 72 h. Mean HPAEC HMOX1 protein (**C**) or mRNA (**D**) were measured with Western blots or qRT-PCR, respectively. Each bar represents the mean ± SE expressed as fold-change vs. CON. * *p* < 0.05 vs. *n* = 3–6. (**E**–**G**) HPAECs were treated with scrambled siRNA or siHMOX1 for 6 h. Selected cells were treated with CON or HEM for 72 h. Mean HPAEC HMOX1 activity was measured with In-Cell ELISA (**E**). Mean HPAEC ET-1 (**F**) or VCAM1 (**G**) levels were measured with qRT-PCR. All bars represent the mean levels relative to GAPDH ± SE expressed as fold-change vs. CON/siHMOX1(-), * *p* < 0.05 vs. CON/siHMOX1(-), + *p* < 0.05 vs. HEM/siHMOX1(-). *n* = 3–4.

HMOX1 is an essential enzyme that degrades heme released during intravascular hemolysis and helps protects endothelial function in SCD. Because HMOX1 levels are associated with a reduced risk of complications like acute chest syndrome in SCD patients, and strategies to increase HMOX1 activity show therapeutic promise for both SCD and PH, we measured HMOX1 levels and activity in vivo and in vitro. As shown in Figure 4, HMOX1 levels are increased in *SS* mouse lungs (Figure 4A,B) and in hemin-treated HPAECs (Figure 4C,D). To determine if loss of HMOX1 modulates endothelial function, HPAECs were transfected with siHMOX1 and treated with hemin. Consistent with data shown in Figure 4A,D, treatment of HPAEC with hemin upregulated HMOX1 activity (Figure 4E) and markers for endothelial dysfunction (Figure 4F,G). Furthermore, we demonstrated that knockdown of HMOX1 not only reduced HMOX1 activity in hemin-treated HPAECs (Figure 4E) but also increased ET-1 expression (Figure 4F) and augmented hemin-induced VCAM1 expression (Figure 4G). These data suggest that hemolysis upregulates HMOX1 and that HMOX1 may play a protective role in hemolysis-induced endothelial dysfunction.

### 3.5. MALAT1 Regulates HMOX1 mRNA and Protein Expression

**Figure 5:**
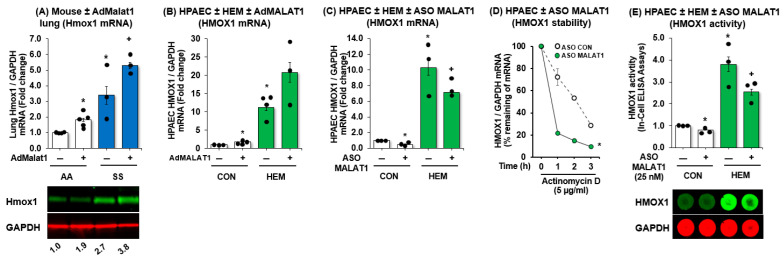
MALAT1 regulates HMOX1 expression and activity. (**A**) *AA* and *SS* mice were infected with AdGFP or AdMALAT1 and 2 weeks later, whole lung homogenates were collected and subjected to qRT-PCR and Western blot analysis. Mean HMOX1 levels examined as fold-change vs. *AA*/AdMALAT1(-). * *p* < 0.05 vs. *AA*/AdMALAT1(-), + *p* < 0.05 vs. *SS*/AdMALAT1(-), *n* = 3–5. (**B**) HPAECs were treated with scrambled AdGFP or AdMALAT1 particles (5 MOI) for 6 h then treated with dimethyl sulfoxide vehicle (CON) or hemin (HEM, 5 µM) for an additional 72 h. Mean HPAEC HMOX1 levels were measured with qRT-PCR. All bars represent the mean mRNA levels relative to GAPDH ± SE expressed as fold-change vs. CON/AdMALAT1(-). * *p* < 0.05 vs. CON/AdMALAT1(-), + *p* < 0.05 vs. HEM/AdMALAT1(-), *n* = 3–4. (**C**,**D**) HPAECs were treated with scrambled anti-sense oligonucleotide (ASO CON) or ASO MALAT1 (25 nM) for 6 h then treated with dimethyl sulfoxide vehicle (CON) or hemin (HEM, 5 µM) for an additional 72 h. Mean HPAEC HMOX1 levels were measured with qRT-PCR (**C**) or HMOX1 activity using In-Cell ELISA (**D**). All bars represent the mean mRNA levels relative to GAPDH ± SE expressed as fold-change vs. CON/AdMALAT1(-). * *p* < 0.05 vs. CON/AdMALAT1(-), + *p* < 0.05 vs. HEM/AdMALAT1(-), *n* = 3–4. (**E**) To inhibit de novo HMOX1 mRNA synthesis, selected cells were treated with 5 μg/mL actinomycin D (in a time-dependent manner). HMOX1 mRNA half-life was determined by comparing to the mRNA level before adding actinomycin D. Error bars represent mean ± SE, * *p* < 0.05 vs. scrambled controls (SCR), + *p* < 0.05 vs. CON/ASO MALAT1(-), *n* = 3.

To assess whether MALAT1 can mediate HMOX1, we overexpressed MALAT1 in vivo and in vitro using adMALAT1. In Figure 5, we showed that MALAT1 overexpression upregulated HMOX1 mRNA and protein in *AA* mice lungs and augmented HMOX1 mRNA and protein expression in *SS* mice lung (Figure 5A, Supplementary Figure S2). In parallel, we showed that MALAT1 overexpression upregulates HPAECs HMOX1 expression but did not further augment the HMOX1 expression in hemin-treated HPAECs (Figure 5B). In contrast, ASO-mediated depletion of HPAEC MALAT1 reduced hemin-induced HMOX1 levels in HPAECs and attenuated hemin-induced HMOX1 expression (Figure 5C). We further found that knockdown of MALAT1 reduced HMOX1 mRNA stability (Figure 5D) and HMOX1 activity (Figure 5E). These findings indicate that MALAT1 regulates the constitutive expression of HMOX1. Taken together, the findings support the postulate that MALAT1 regulates endothelial function via HMOX1 activation, a previously undescribed pathobiological pathway in SCD-related endothelial dysfunction and PH.

## 4. Discussion

In this study, we present data supporting a functional role of MALAT1 in SCD-PH pathogenesis. Using lncRNA microarray and confirmed by quantitative PCR, we showed that MALAT1 is upregulated in a chronic hemolysis environment including in *SS* mouse lungs and hemin-treated HPAECs. Further, depletion of MALAT1 promoted and exacerbated hemin-induced endothelial dysfunction as reflected by an upregulation of both ET-1 and VCAM1. Conversely, MALAT1 overexpression suppressed ET-1 and VCAM1 expression and attenuated hemin-induced ET-1 and VCAM1 expression in HPAECs. Moreover, in vivo MALAT1 overexpression decreased RVSP, RVH, endothelial proliferation, and markers of endothelial dysfunction. To understand the mechanism underlying the protective effect of MALAT1 in SCD, we identified HMOX1 as a candidate target for MALAT1. We show that HMOX1 levels increase in *SS* mouse lungs and hemin-treated HPAECs. Similarly to MALAT1, suppression of HMOX1 is associated with an increase in endothelial cell dysfunction suggesting that HMOX1 and MALAT1 affect HPAECs in a similar fashion. In parallel, we show that MALAT1 mediates HMOX1 as MALAT1 overexpression upregulated HMOX1 while inhibition of MALAT1 reciprocally suppressed HMOX1. Finally, inhibition of MALAT1 is associated with HMOX1 RNA instability. Currently, our ongoing studies are focused on investigating the potential indirect regulation of HMOX1 by MALAT1, possibly through intermediary transcriptional or post-transcriptional mechanisms. Collectively, these data support our hypothesis that the MALAT1–HMOX1 axis plays an important role in hemolysis-induced endothelial dysfunction and, ultimately, hemolysis-associated pulmonary hypertension as seen in SCD lungs.

Long non-coding RNAs (lncRNA) are abundantly expressed in all tissues and participate in cellular functions and disease pathogenesis [36]. lncRNAs can regulate gene and protein expression by acting as gene activators or repressors, mRNA stabilizers, miRNA precursors and inhibitors, and direct interaction with DNA or protein [36]. LncRNA MALAT1 consists of ~8000 bp and is highly conserved and abundantly expressed in various cells and tissues including in PAECs [23]. MALAT1 polymorphisms either increase the risk of developing PH (i.e., rs619586A>G single nucleotide polymorphism) or are protective (i.e., G variant) [24]. The protective effect of the MALAT1 G variant is through its function as a competitive endogenous RNA for miR-214, which leads to an inhibition of vascular EC proliferation and migration [24]. Several lncRNAs have been implicated in PH pathogenesis including MALAT1. Specifically, MALAT1 is highly expressed in hypoxia-induced pulmonary artery smooth muscle cells (PASMCs) and PAECs [37]. In this study, we utilized a microarray for lncRNAs to identify possible novel diagnostic or therapeutic targets and identified MALAT1 as the only lncRNA that was consistently upregulated in *SS* lungs. As vascular EC dysfunction is one of first triggers leading to development of PH [38], we assessed the function of MALAT1 on EC dysfunction in a chronic hemolysis model as seen in people with SCD. As presented in this report, modulation of MALAT1 has an inverse relationship with markers of EC dysfunction. Further, overexpression of MALAT1 protected EC against hemin-induced EC dysfunction. These findings support the functional significance of MALAT1 in hemin (chronic hemolysis)-induced EC dysfunction. Recent advances in ASO technology (e.g., LNA gapmer) and siRNA-based strategies have been successfully used to knock down MALAT1 in cells and animal models, resulting in meaningful biological effects such as reduced proliferation, migration, and metastasis in cancer models, and altered vascular or cardiac phenotypes in preclinical studies [37,39,40,41]. Since our current study suggests that MALAT1 overexpression exerts protective effects, PH therapeutic strategies should instead focus on promoting MALAT1 expression. First, recent evidence indicates that m^6^A (N^6^-methyladenosine) RNA modification can modulate MALAT1 expression. For instance, METTL3-dependent methylation has been reported to enhance MALAT1 stability and expression in human cancer cells [42]. Second, emerging evidence suggests that microRNAs (miRNAs) serve as functional regulators of gene expression across multiple critical cellular pathways [16,17,18,19]. Our preliminary data indicate that targeting reciprocal repressors—specific miRNAs and MALAT1—may have therapeutic potential in SCD-PH. In particular, inhibition of certain miRNAs that suppress MALAT1 expression appears to increase MALAT1 levels and favorably modulate a spectrum of pathobiological responses in SCD-PH. Third, to further verify this mechanism, chromatin immunoprecipitation (ChIP) assays in HPAECs will be conducted to confirm direct PPARγ binding to the MALAT1 promoter in a future study [33,43,44].

The pathobiology of SCD-PH involves abnormal proliferation of pulmonary vascular wall cells, vascular remodeling, and muscularization of small pulmonary vessels [45]. These structural and functional alterations in the pulmonary vasculature increase pulmonary vascular resistance and promote progressive right-sided heart failure and death. Sickle cell mice spontaneously develop PH as shown by elevated pulmonary artery pressure, decreased cardiac output, and right-heart failure [46]. Furthermore, our group and others showed that *SS* mice have evidence of intravascular hemolysis, which induces EC pro-inflammatory responses, including IL-8, IL-6, VCAM-1, and ET-1 levels [35,47]. Given that MALAT1 is protective against EC dysfunction, we assessed the function of MALAT1 in *SS* mouse lungs. Consistent with the in vitro findings, we show that overexpression of MALAT1 in *SS* mouse lungs attenuated RVSP, RVH, vascular remodeling, and markers of EC dysfunction. These data suggest that therapeutic upregulation of MALAT1 could prevent or possibly reverse histological and physiological changes consistent with SCD-PH in *SS* mouse lungs.

A hallmark of SCD is intravascular hemolysis leading to a release of free heme (hemin) leading to disrupted EC function and exacerbated oxidative stress [48,49]. HMOX1 is an essential enzyme that degrades heme released during intravascular hemolysis, protects EC function in SCD, and is implicated in PH [50,51,52]. HMOX1 activity promotes antioxidant, cytoprotective, and heme catabolic pathways as well as protects endothelial function [52]. Reduced HMOX1 expression has been observed in the lungs of patients with PH [53] and hypoxia exacerbates right ventricular dilatation in *Hmox1* KO mice [26]. Conversely, alveolar epithelial cell-targeted human HMOX1 overexpression decreased hypoxia-induced PH [27]. Intriguingly, increases in HMOX1 were observed in neutrophils, mononuclear cells [28], and tissues in patients and transgenic mice with SCD [29]. In this study, we show that lungs from *SS* mice express higher levels of HMOX1 compared to littermate control lungs and hemin treatment induces HMOX-1 expression in HPAECs. Further, inhibition of HMOX1 expression exacerbates EC dysfunction in hemin-treated HPAECs. These findings are consistent with previous reports suggesting that HMOX1 is induced in the setting of hemolysis, and the lack of HMOX1 promotes EC dysfunction.

The regulatory networks involving various types of non-coding RNAs have been increasingly explored. These non-coding RNAs include miRNAs and lncRNAs, which can form a complex regulatory network. microRNAs (miRNAs or miR) are endogenous, non-coding, single-stranded RNAs of ~22 nucleotides that function as post-transcriptional gene regulators [54]. Accumulating evidence demonstrates that dysfunction of miRNAs participates in SCD pathogenesis [55]. These findings suggest that miRNAs can be used as functional regulators via inducing or inhibiting other miRNAs during disease progression in SCD. Several miRNAs have been shown to regulate HMOX1, including miR-377 in combination with miR-217 [56], let-7 [57], miR-155 [58], miR-135a, miR-135b [59], miR-872 [60], and miR-107 [61]. However, less is known about the role of lncRNA in HMOX1 regulation. Given the reciprocal changes between MALAT1 and HMOX1 in *SS* mouse lungs, we speculated that MALAT1 might play a regulatory role on HMOX1. Consistent with our speculation, we showed that overexpression of MALAT1 upregulated HMOX1, while inhibition of MALAT1 resulted in suppression of HMOX1 in mouse lungs and HPAECs. We are currently investigating the potential indirect regulation of HMOX1 by MALAT1 through intermediary transcriptional or post-transcriptional mechanisms as part of our ongoing research, which also includes in-depth bioinformatics analyses of our lncRNA microarray.

In summary, the current study identifies novel functions of the MALAT1–HMOX1 axis in the pathogenesis of SCD-PH. The outcomes of the proposed studies will advance our understanding of lncRNA MALAT1 in SCD-PH and provide new therapeutic targets for treating people with SCD-PH and related diseases.

## Figures and Tables

**Table 1:** Human and mouse primer sequences.

**Human**	**Forward (5′-3′)**	**Reverse (5′-3′)**
GAPDH	GCCCAATACGACCAAATCC	AGCCACATCGCTCAGACAC
ET-1	TCTCTGCTGTTTGTGGCTTG	GAGCTCAGCGCCTAAGACTG
VCAM1	TGCACAGTGACTTGTGGACAT	CCACTCATCTCGATTTCTGGA
HMOX1	GGGTGATAGAAGAGGCCAAGA	AGCTCCTGCAACTCCTCAAA
MALAT1	GGGGGAGTTTTCAGTATTTTTTTTTG	TACACCTTGAGTCATTTGCCTTTAGG
**Mouse**	**Forward (5′-3′)**	**Reverse (5′-3′)**
GAPDH	AGCTTGTCATCAACGGGAAG	TTTGATGTTAGTGGGGTCTCG
ET-1	CTGCTGTTCGTGACTTTCCA	TCTGCACTCCATTCTCAGCTC
VCAM1	TCTTACCTGTGCGCTGTGAC	ACTGGATCTTCAGGGAATGAGT
HMOX1	AGGGTCAGGTGTCCAGAGAA	5′-CTTCCAGGGCCGTGTAGATA
MALAT1	CACTCTGGGAATGTTTTTGG	TGTCGAAAAGAGGTGGTGTG

## Data Availability

Data is contained within the article or Supplementary Material.

## Supplementary Materials

The following supporting information can be downloaded at https://www.mdpi.com/article/10.3390/cells15020154/s1: Figure S1: The efficiency of adenoviral-mediated MALAT1 overexpression in vivo and in vitro, Figure S2: MALAT1 regulates HMOX1 protein levels.; Table S1: Differentially expressed lncRNAs between *SS* and *AA* mouse lungs.

## Abbreviations

The following abbreviations are used in this manuscript:

PH	Pulmonary hypertension
SCD	Sickle Cell Disease
MALAT1	Metastasis-associated lung adenocarcinoma transcript 1
SS	Sickle cell
HMOX1	Heme oxygenase-1
lncRNAs	Long non-coding RNAs
AA	Littermate controls
HPAECs	Human pulmonary artery endothelial cells
VCAM1	Vascular endothelial cell adhesion molecule 1
RVH	Right ventricular hypertrophy
ASO	Antisense oligonucleotide
ET-1	Endothelin-1
HEM	Hemin
RVSP	Right ventricular systolic pressure
miRNAs	microRNAs
EC	Endothelial cells
GFP	Green fluorescent protein
