# Supplementary figures and images for: Long Non-Coding RNA MALAT1 Regulates HMOX1 in Sickle Cell Disease-Associated Pulmonary Hypertension
Source: Cells. 2026 Jan 15;15(2):154. doi: 10.3390/cells15020154 (PMC12839230; doi:10.3390/cells15020154)

Supplementary Figure S1

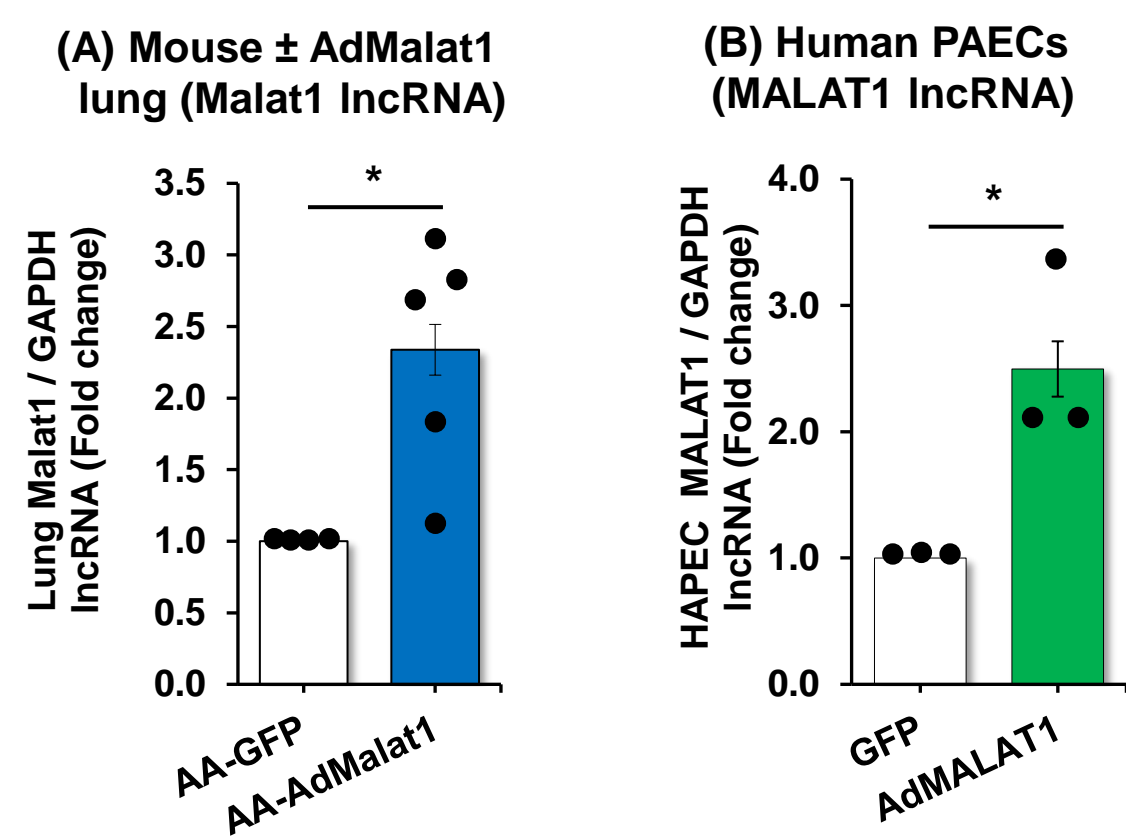

Supplement: Supplementary file 1 [file cells-15-00154-s001.zip › Supplementary Figure S1. pptx.pdf]

Supplementary Figure S2

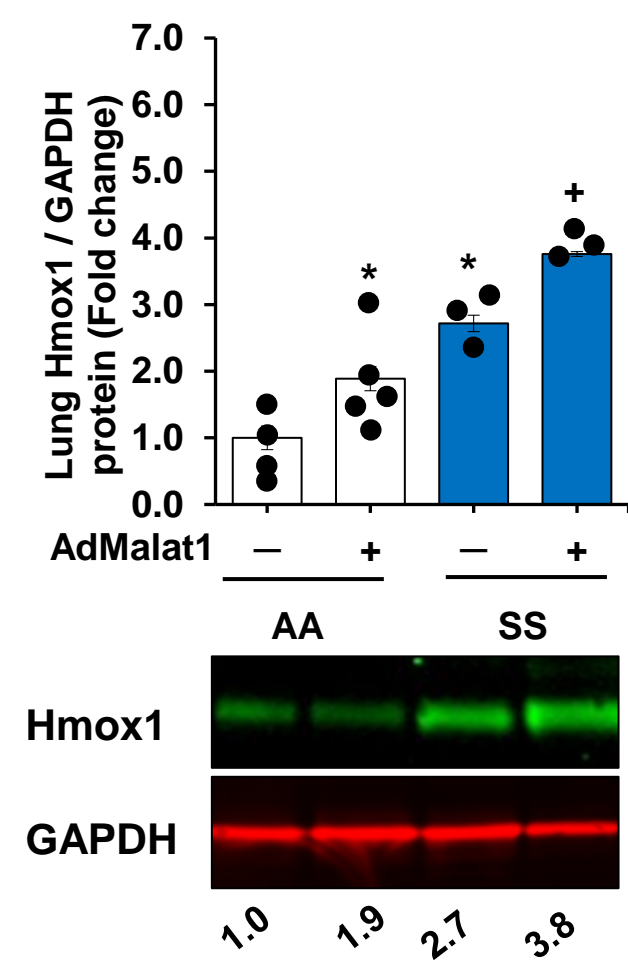

Supplement: Supplementary file 1 [file cells-15-00154-s001.zip › Supplementary Figure S2. pptx.pdf]
